# Illusions of having small or large invisible bodies influence visual perception of object size

**DOI:** 10.1038/srep34530

**Published:** 2016-10-06

**Authors:** Björn van der Hoort, H. Henrik Ehrsson

**Affiliations:** 1 Department of Neuroscience, Karolinska Institute, Retzius väg 8, 17177, Stockholm, Sweden

## Abstract

The size of our body influences the perceived size of the world so that objects appear larger to children than to adults. The mechanisms underlying this effect remain unclear. It has been difficult to dissociate visual rescaling of the external environment based on an individual’s visible body from visual rescaling based on a central multisensory body representation. To differentiate these potential causal mechanisms, we manipulated body representation without a visible body by taking advantage of recent developments in body representation research. Participants experienced the illusion of having a small or large invisible body while object-size perception was tested. Our findings show that the perceived size of test-objects was determined by the size of the invisible body (inverse relation), and by the strength of the invisible body illusion. These findings demonstrate how central body representation directly influences visual size perception, without the need for a visible body, by rescaling the spatial representation of the environment.

Philosophers and theoretical psychologists have suggested an important role for the size of our body in our visuospatial perception of the external environment^1^,^2^. In this embodied view of vision, our body is thought to serve as a “fundamental ruler” in visuospatial perception whereby the size of external objects are perceived in relation to the size the body of the observer.

This theory has been tested using body-ownership illusions^3^,^4^ in which participants experience an artificial body that they see from a first-person perspective as their own, so that tactile and proprioceptive sensations originate from this artificial body^5^. Inducing such an illusion requires synchronous touching of the participant’s physical body and the artificial body at corresponding body-parts and in the same direction, similar to the classic rubber hand illusion^6^,^7^. By systematically changing the size of the artificial body, it has been shown that having a small artificial body increases the perceived size and distance of objects, while a large artificial body reduces the perceived size and distance of objects^3^,^4^. Similar perceptual changes have been described for a change in hand size^8^,^9^ and in virtual reality experiments where participants embody a virtual hand^10^ or child-body^11^. In this “own-body-size effect”, the sensation of body-ownership appears to be critical because the effect disappears when ownership is disrupted by the application of asynchronous visuotactile stimulation. Furthermore, this own-body-size effect does not require that the artificial body is visible once the ownership illusion is induced^4^.

Nevertheless, the critical question remains: *how* does having a different sized body cause visual perception to be altered? Does the artificial body simply act as a very strong visual familiar size cue that rescales the visible environment when the sensation of body-ownership is present? Or is it a central representation of the body that directly influences the visuospatial perception of the environment without the need for a visible body? Here, we directly test the latter possibility, i.e. that a dynamic spatial realignment of visual information to somatosensory information is what elicits the “own-body-size” effect, while we exclude the possibility that a visible body acts as a familiar size cue. To this end, we combined the “Barbie-doll illusion^3^” with the recently reported “Invisible body illusion^12^” to create illusions of participants having small or large invisible bodies and to investigate the consequent effects on visual object size perception. In the invisible body illusion, participants observe a brush ‘touching’ in the air. When participants feel a touch that is synchronously and spatially congruent with these observed touches, they perceive that the observed and felt touches are the same and that they originate from their body, which is now invisible. Crucially, we manipulate the size of the invisible body by introducing a spatial misalignment of depth, i.e., the brush that they see can either be closer by (60–80 cm: small invisible body) or further away (300–400 cm: large invisible body) than the touches that they feel (135–180 cm). Similarly to other body-ownership illusions, this initial misalignment is thought to be resolved by recalibrating the different senses and the central body representation in a way that this multisensory conflict is minimized^13^ because the human brain has a tendency to bind information from different senses together into meaningful percepts^14^,^15^,^16^. This should lead participants to perceive the felt touch and observed touch to be the same and to experience illusions of owning small or large invisible bodies. Consequently, this recalibration is predicted^4^ to alter the perception of external space because the own body defines the body-centered spatial reference frame, which is a fundamental aspect of the external space representation. The construction of external space involves the transformation of body-centered reference frames into allocentric reference frames^17^. Thus, changing the perceived size of one’s own body by multisensory illusions should have profound effects on spatial perception. For such multisensory recalibrations, the rationale is that if the observed touch is further away than the felt touch (i.e. in the large invisible body illusion), the visuospatial representation of external space would need to be shrunken to eliminate conflict and realign it with the tactile event. This would cause any object in the room to be perceived as being smaller. By contrast, if participants see the brush moving close-by (i.e. in the small invisible body illusion), the visual representation of the external space would need to be enlarged and, thus, external objects would be perceived as being larger.

In the current study, participants estimated the size of different objects (10 cm, 20 cm, 30 cm), during four different visuotactile conditions: small-synchronous, large-synchronous, small-asynchronous, and large-asynchronous. We expected participants to experience the illusion of owning an invisible body when the observed touch and the felt touch are synchronous but not when they are asynchronous. Furthermore, we predicted the size estimations to be largest when participants experience the small body illusion (i.e. in the ‘small-synchronous’ condition), and smallest when they experience the large body illusion (i.e. in the ‘large synchronous’ condition). In the asynchronous conditions (i.e. ‘small-asynchronous’ and ‘large-asynchronous’) we expected the invisible body illusion to be eliminated and the effect on size estimations to disappear.

## Results

### Size estimations

**Figure 1:**
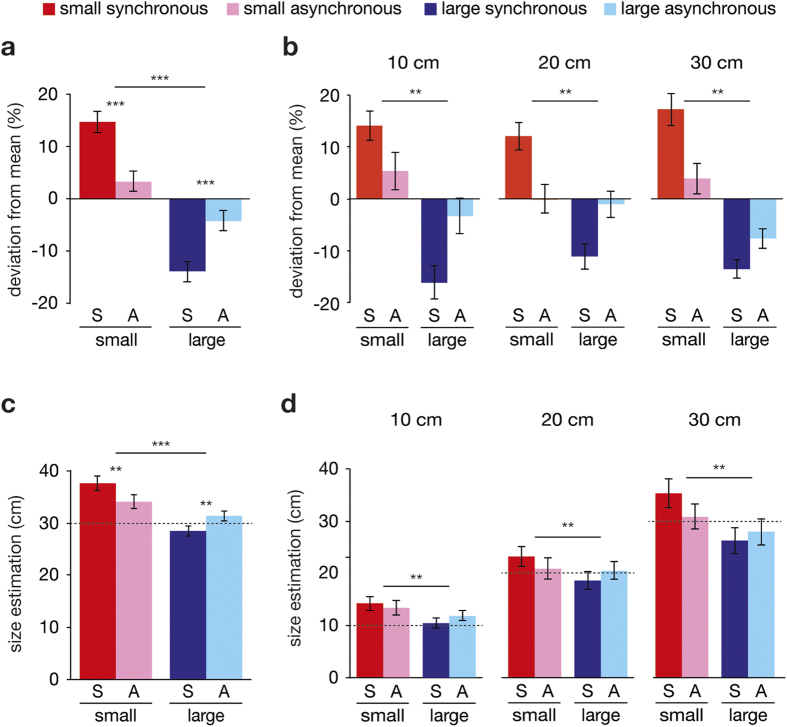
Size estimations. Size estimations for the four different conditions as a deviation from individual’s means plotted for the average (**a**) and for each object separately (**b**), and size estimations in cm for the normalized average (**c**) and for each object and (**d**). Bars indicate means and error bars indicate SE. Significance stars between two bars indicate p-value from paired t-test, and significance stars above horizontal line indicate p-value from interaction-effect of ANOVA (**p < 0.01; ***p < 0.001).

For the main experiment, we detected an effect of the invisible body size on size estimations (small body > large body: F (1, 21) = 37.231, p < 0.001, *η*^***2***^ = 0.64), and we found a significant interaction between invisible body size (small versus large) and the synchronicity of the touches (synchronous versus asynchronous) (F (1, 21) = 27.689, p < 0.001, *η*^***2***^ = 0.57) (Fig. 1a). These effects are in line with our expectations. The size estimations were significantly larger in small-synchronous compared to small-asynchronous (t (21) = 4.117, p < 0.001, *d* = 0.88) and significantly smaller in large-synchronous versus large-asynchronous (t (21) = −3.839, p = 0.001, *d* = −1.06). As expected, the largest difference was found between small-synchronous and large-synchronous (t (21) = 7.904, p < 0.001, *d* = 1.69). Thus, participants estimated objects to be larger when their invisible body was small, and vice versa, participants estimated objects to smaller when their invisible body was large. Crucially, these effects disappeared when the illusion of owning an invisible body was disrupted by asynchronous visuotactile stimulation, despite the visual information being identical to that in the synchronous conditions. As expected these effects did not differ between different object sizes (F (2, 20) = 0.044, p > 0.20). Each object size showed the expected interaction effect (10 cm: F (1, 20) = 14.242, p = 0.001; 20 cm: F (1, 20) = 14.700, p = 0.001; 30 cm: F (1, 20) = 8.884, p = 0.007) (Fig. 1b).

All the results described above were reproduced when we analyzed the raw data (in cm) before its transformation to percentages as deviations from the means (see Method). The ANOVA revealed a significant main effect of body size (F (1, 21) = 21.304, p < 0.001, *η*^***2***^ = 0.50), and a significant interaction effect with visuotactile synchronicity (F (1, 21) = 23.751, p < 0.001, *η*^***2***^ = 0.53) (Fig. 1c). The paired t-tests revealed significant differences between small-synchronous and small-asynchronous (t (21) = 3.572, p = 0.002, *d* = 0.77), and between large-synchronous and large-asynchronous (t (21) = −3.912, p = 0.001, *d = *−0.84). There was no effect of object size (F (2, 20) = 0.030, p > 0.20), and each object size showed the expected interaction effect (10 cm: F (1, 20) = 14.143, p = 0.001; 20 cm: F (1, 20) = 9.807, p = 0.005; 30 cm: F (1, 20) = 8.629, p = 0.008) (Fig. 1d).

### Illusion questionnaire

**Figure 2:**
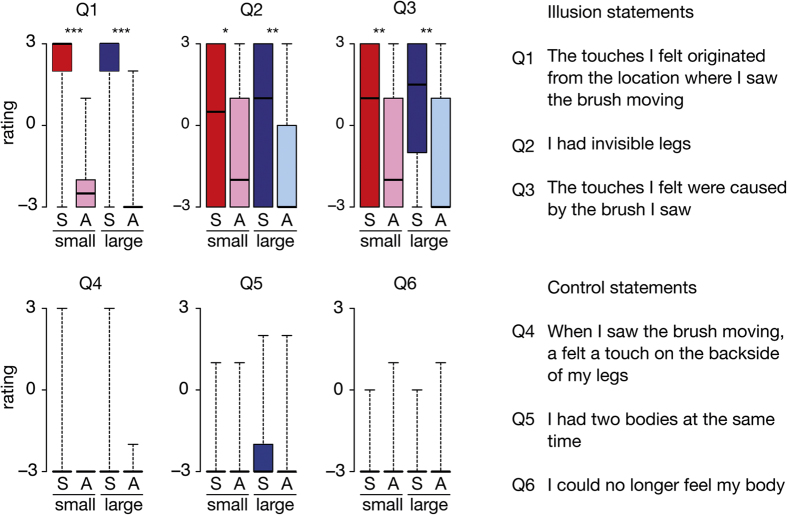
Questionnaire data. Boxplots of the ratings for each statement on the questionnaire for each condition. Significance stars between two boxplots indicate the p-value of the associated Wilcoxon signed rank test (*p < 0.05, **p < 0.01, ***p < 0.05).

**Figure 3:**
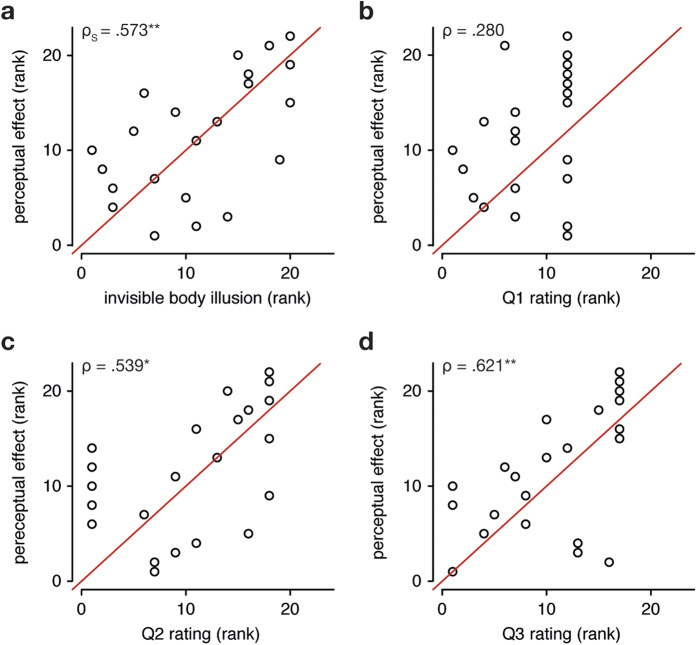
Correlation between invisible body illusion and perceptual effect. Correlation plots with the ranks of the total illusion score (**a**), the ranks of Q1 rating (**b**), the ranks of Q2 rating (**c**) and the ranks of Q3 rating (**d**) on the x-axes, and the ranks of the perceptual effect on the y-axis (own-body size effect on size estimation, see methods for details). The value in the top-left corner of each plot indicates Spearman’s rho for nonparametric correlations, and the significance stars indicate the associated p-value (*p < 0.05, **p < 0.01) after a Bonferroni correction.

To assess the strength of the illusion for each condition, participants rated each of six questionnaire statements, of which three captured the invisible body illusion (see Method and Fig. 2). The questionnaire data was acquired after the size estimation experiment and therefore could not have influenced the results described above. Participants affirmed the three illusion statements more in the small-synchronous condition compared to the small-asynchronous condition (Q1: Z = 3.920, p < 0.001, *r* = 0.59; Q2: Z = 2.359, p = 0.018, *r* = 0.36; Q3: Z = 2.917, p = 0.004, *r* = 0.44; Fig. 3) but their ratings did not differ between these two conditions for the control statements (Q4: Z = −1.414, p = 0.16; Q5: Z = −0.351, p > 0.20; Q6: Z = −0.213, p > 0.20). The same pattern appeared when comparing large-synchronous with large-asynchronous: higher ratings on illusion statements in the large-synchronous condition (Q1: Z = 4.091, p < 0.001, *r* = 0.62; Q2: Z = 3.311, p = 0.001, *r* = 0.50; Q3: Z = 2.642, p = 0.008, *r* = 0.40) but no difference between these conditions for the control statements (Q4: Z = 0.000, p > 0.20; Q5: Z = −1.380, p = 0.17; Q5: Z = −0.272, p > 0.20). Thus, the small invisible body and the large invisible body illusions were elicited, as we had expected.

Despite this overall effect, participants varied substantially in the reported strength of these illusions (Fig. 2, Q2 and Q3). This allowed us to investigate the effect of participants’ illusion strength on their object size estimations through a correlation analysis (see Method).

### Correlation analysis: illusion score x perceptual effect

We predicted that participants with a stronger invisible body illusion would have a stronger effect on their visual object size perception. Indeed, the illusion score was positively correlated with the perceptual effect (ρ_S_ = 0.573, p = 0.005) (Fig. 3a). Thus, the more participants experienced the illusion of owning a small or large invisible body, the stronger the body-size effect on their perception was. In addition, we found that this correlation seemed to be driven by the ratings of Q2 and Q3, i.e. the two illusion statements that showed the largest variability (Q2: ρ_S_ = 0.539, p = 0.029; Q3: ρ_S_ = 0.621, p = 0.006; Bonferroni corrected) (Fig. 3c,d). The ratings on Q1 did not significantly correlate with the perceptual effect (Q1: ρ_S_ = 0.279, p > 0.20; Bonferroni corrected), which is probably due to the relatively low variability of the ratings for this illusion statement (Fig. 3b).

## Discussion

Our findings show that when participants observe a brush moving nearby (40–80 cm) and synchronously with strokes that they feel on their real (but unseen) body, they experience the illusion of having a small invisible body and they perceive test-objects to be larger. In contrast, when participants see a brush moving far away (200–400 cm) and synchronously with strokes that they feel on their real body, they experience the illusion of having a large invisible body and they perceive test objects to be smaller. Importantly, these effects are disrupted when the observed strokes are asynchronous with the felt strokes. In addition, our results show that, across participants, the stronger the invisible body illusion the stronger the own body size effect on visual size perception.

Our findings refute the hypothesis that elicitation of the own-body-size effect occurs through a visual recalibration of external space that is based on a visible body of a certain size, i.e., that the body in view acts as a familiar size cue. Instead, our results support the hypothesis that the spatial realignment of visual perception and tactile perception that is necessary for body-ownership illusions^5^,^13^,^18^ is also responsible for the own-body-size effect. Specifically, if the observed touch is further away than the felt touch (large invisible body illusion), the visually perceived external space must be shrunk to perceive the touches to arise from the same location and to experience body-ownership, which causes test-objects in such an environment to shrink accordingly. By contrast, when the observed touch is closer than the felt touch (small invisible body illusion), external space must be enlarged to re-align the different senses and induce body-ownership, which causes the test-objects to appear larger. Importantly, this perceptual change of objects in external space occurs irrespective of the distance from the observer’s body^4^. To be clear, the size of body space and the perceived size of external space are interdependent and perceived relative to each other, such that when the size of body space changes relative to external space, the perceived size of external space changes automatically in the opposite direction. Taken together, our findings demonstrate that visual object size perception is directly influenced by a central body representation via an automatic link between body space (i.e. the space occupied by one’s body) and external space, without the need for seeing the body.

In our study the central body representation was manipulated through visuo-tactile synchrony. However, ownership of a body(-part) can also be induced through visuo-motor synchrony^19^,^20^,^21^,^22^. In virtual reality studies that make use of such visuo-motor synchrony to induce ownership of an avatar, the size of a virtual body(-part) has been shown to have an effect on visual perception^10^,^11^ similar to the one described in present study. Because of the visibility of the virtual body in these virtual reality experiments, the body could theoretically work as a visual relative size cue. However, we hypothesize that these effects are caused by a recalibration mechanism that is similar to the one described for the current study. When visuo-motor synchrony leads to ownership of a small avatar, the shrunk body space recalibrates external space causing it to be perceived as larger. Thus, we hypothesize that the link between body space and external space will cause a recalibration of visual size perception every time the body for which ownership is felt changes in size, irrespective of the method used to induce such ownership.

Another line of embodied vision research focuses not on the size of the body *per se* but on the action possibilities and action capabilities of that body. For example, increasing the body’s action radius through the use of a tool makes distances appear shorter^23^. In addition, the perceived size of an aperture decreases when the arms are spread sideways because the resulting body posture does not allow walking through the aperture^24^. Changing the action capabilities of a body can also alter visual perception of external space, for example wearing a heavy backpack makes hills appear steeper^25^ and increasing sugar blood levels can make hills appear less steep^26^. Furthermore, being skilled in a certain task can change visual perception: skilled golfers perceive the hole to be larger than less-skilled golfers^27^, and skilled baseball players perceive the ball to be larger than less-skilled players^28^. Thus, visual perception of size and distance is not only affected by the size of our body, but also by what that body can do and how skilled it is.

It remains unclear how the effects of body size and body capabilities on visual perception relate to each other. One possibility is that they share the same mechanism: the size of a body defines the capabilities of that body such that a small body limits action possibilities and increases the effort associated with a particular action. According to this view, all body scaled effects on visual perception can be attributed to changes in action possibilities and action capabilities of the body. However, another possibility is that body size has a more fundamental effect on visual perception^4^, and that any additional effects of posture, physiological state, and skills of that body, and the tools it uses, are secondary. According to this view the central representations of body space, near-personal external space and external space far from the body are intimately linked so that changing the former automatically changes the latter two. Such central realignment of spatial representations would affect action because goal-directed actions towards environmental objects require planning in external coordinate systems^29^,^30^,^31^.

Some of the aforementioned studies on the role of affordances on visual perception have been criticized for not controlling for task demand effects. For example, the effects of backpacks on slope estimation disappear when participants are told to ignore the backpack^32^. Such task demand effects are unlikely to apply to the method that is used in the current study. Participants had no prior experience with experiments on body-ownership illusions, and therefore could not have had expectations as to how this would affect their visual perception. Furthermore, the direction of the effect of synchronous visuotactile stimulation on visual size perception is opposite for the small body and the large body. Such an interaction effect, solely based on the synchronicity of visuotactile stimuli, seems unlikely to be due to task demands or expectancy effects.

Another consideration is the possible use of visual imagery during the invisible body illusion. The concern is that the strokes in empty space, or the body illusion itself, could encourage the participant to start mentally imagining seeing a body, and that it is this mental image that causes the own body size effect rather than the body illusion *per se*. We do know that visual imagery can affect visual perception of size^33^ and that the functional relationship between imagery and perception is intimate^34^,^35^. However, it remains unclear if synchronous visuotactile stimulation induces mental visual imagery of a body, and if it does so to a greater extent than the asynchronous visuotactile stimulation. Future experiments are needed to investigate the possible role of visual imagery in the invisible body illusion.

To summarize, this study is first to show that there is no need for a visible body in the own-body size effect. The inverse scaling of visual perception by body size is not mediated by visual information from the body but instead requires the alignment of a non-visual body representation and the visual representation of external space. This finding advances our understanding of the processes that mediate embodied vision.

## Methods

### Ethics statement

Participants provided written informed consent prior to participating in this study. The methods were approved by the Regional Ethical Review Board of Stockholm, and the experiments were carried out in accordance with these approved guidelines.

### Participants

We recruited 22 naïve, healthy adult participants by placing advertisements on the campus of Stockholm University. Participants received a cinema voucher in exchange for their participation. The required group size was assessed through a power analysis for the planned paired t-tests between synchronous and asynchronous conditions, with α = 0.05 and β = 0.95. Previous studies have shown very large effect sizes for the effect of body-ownership on visual perception (*d* > 0.80)^3^,^4^. However, since the effect of ownership has never been tested with the invisible body illusion we made a relatively conservative prediction of the effect size (medium-large effect size: *d* = 0.65), which requires a sample size of 21 participants to reach statistical significance (power analysis was performed with G*Power 3.1)^36^.

**Figure 4:**
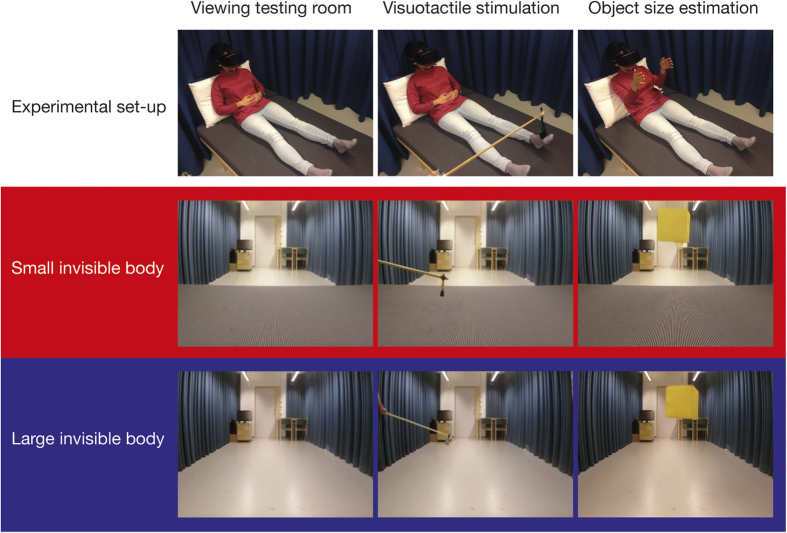
Experimental set-up and video stimuli. A participant wearing the head-mounted displays (HMDs) during different stages of an individual trial is illustrated in the top row. The middle and bottom rows show still-frames from the video stimuli displayed in the HMDs during the small invisible body conditions (middle row) and the large invisible body conditions (bottom row).

Participants wore a set of Oculus Rift HMDs (Oculus VR, Menlo Park, California, United States) in which they watched a 3D movie of a room in which a brush was moving. Movies were recorded with two CamOne Infinity cameras (CamOneTec, Delbrück, Germany) placed 85 mm apart and 100 cm from the floor for each experimental condition. The identical “eye-height” (100 cm) in each condition excluded the possible confounding factor of visual perspective on object-size perception. In each movie, the testing room was visible, which included a desk, chair, and door (Fig. 4). These familiar objects would be predicted to attenuate the effects of the experimental conditions but was included to enhance the realism and ecological validity of the setup (in line with van der Hoort *et al*. 2011). The observed brush stroked in four different locations of empty space, which correspond to the lower legs and feet of a small and large invisible body. The brush moved into participants’ field of view to approach the invisible body and make three strokes at one of four locations before it moved back out of the field of view. In the *small body* conditions, a small brush (2 cm wide) was moved between 50 and 80 cm from the cameras, and 5–10 cm above a bed that was placed in front of the cameras. A bed was included to maintain constant “eye-height” across conditions and, consequently, to control for the effect of visual perspective. In the *large body* conditions, a large brush (10 cm wide) was moved between 250 and 400 cm from the cameras and 25–50 cm above the floor. The sizes of the small and large brushes were selected so that they were proportional to the distance and height of the brush-stimuli to match the width of the observed brush with the width of the felt brush.

### Tactile stimulation and visuotactile conditions

In each experimental condition, participants were touched with a medium sized brush (5 cm wide) on their legs and feet in a manner proportional to the touches they observed (see above). The head of the participant was tilted in a way that, while lying down, they could see the lower part of their body but still see the entire testing room. Therefore, visuotactile stimulation was restricted to the lower limbs. Earlier studies have shown that this mode of stimulation can elicit full-body illusions^4^,^37^. The brush strokes were applied to the lower legs and feet. The applied brush strokes were always in the same direction as the observed strokes, and on the corresponding location of the “invisible body”. However, these felt brush strokes could be synchronous or asynchronous with the observed touches, yielding four conditions in a two-by-two design: *small-synchronous, small-asynchronous, large-synchronous*, and *large-asynchronous*. Importantly, apart from the distance of the observed touches, all other parameters were constant, i.e., the size and distance of target objects, the height of the camera, the testing room, and tactile events.

### Procedure

Participants first received instructions in a separate waiting room. In the waiting room, participants were blindfolded before they were led to the testing room, which ensured that participants did not see the experimental set-up or the various objects in the room. Participants were lying down on a bed with their head tilted forward at a 30-degree angle and were fitted with HMDs. Prior to the main experiment, participants estimated the size of three consecutive target objects (10, 20, and 30 cm) without having observed or felt a brush (Fig. 4, left and right column). These control trials were included to ensure that the presence of the bed in the small invisible body conditions did not have an effect on visual perception of object size (t (21) = 1.150, p > 0.250). The main experiment consisted of four blocks: one for each condition. Each block consisted of three trials. The first trial began with 120 seconds of visuotactile stimulation (see previous paragraph) after which a target object was presented at a distance of 150 cm (Fig. 4). After the target object moved out of view, participants bimanually indicated its perceived size by holding out their hands palm-to-palm, and the experimenter manually measured the distance between their hands. The reason to prefer this method is that bimanual size estimations show less variability between participants and within participants compared to verbal size estimations^3^, and therefore are a more reliable and accurate measure. The participants, who were still wearing the HMDs, could not see their hands during this procedure. After this object size estimation, the next trial began with another period of visuotactile stimulation (60 s) in order to keep the illusion from wearing off. Object sizes were 10, 20, and 30 cm, and they were presented once for each condition, yielding a total of twelve trials (3 object sizes x 4 experimental conditions). The order of object sizes was randomized across conditions. The colors of target objects differed in each of the twelve trials, which prevented participants from recognizing an object from a previous trial. The order of conditions was randomized between participants. After these twelve trials, the subjective experience of the invisible body illusion was assessed. Participants rated their subjective experience of the body illusion for each condition on a questionnaire after a period of visuotactile stimulation (120 s) for each of the four conditions. Thus, the questionnaire data were always collected after the main object estimation task and, therefore, could not have influenced the results. The questionnaire consisted of three statements to test different aspects of the illusion (Q1-Q3) and three control statements that controlled for task compliance and expectancy effects (Q4-Q6) (adopted from a previous study^12^) in a random order. For each statement, participants indicated to what extent they agreed or disagreed on a seven point Likert scale in which −3 indicated “strongly disagree”, 0 indicated ”uncertain”, and +3 indicated “strongly agree” (Fig. 2 for specific statements).

### Data and statistics

For the size estimation data, a weighted average was taken across the three trials for each condition by standardizing all estimations to the 30 cm object, yielding a total of four averages per participant. For each participant, we calculated the deviation from the mean for each of these conditions as a percentage, by dividing participants’ size estimations by the mean of all their estimations. These percentages reflect the rescaling of visual perception, which is more informative than the difference in cm between conditions. For example, a change in size-estimation from 10 cm to 20 cm means that an object had doubled its size but a change from 50 cm to 60 cm implies a relatively small increase in size although the increase in “raw” cm is identical. This problem can be dealt with by transforming the data this way. In addition, this normalization of the data removes a large part of the inter-subject variability that is independent of our experimental conditions. This is especially useful for between-subject correlation analyses (see below). Moreover, it leads to a better estimation of effect sizes, especially given the large standard deviation of participants’ means of the raw data (see Results). We performed Kolmogorov–Smirnov tests to assure that the new distribution after this transformation did not violate assumptions of normality. We analyzed the main effects for invisible body size (small versus large) and synchronicity (synchronous versus asynchronous) and their interaction using a repeated-measures ANOVA and their effect size were calculated as eta-squared (η^**2**^). Differences between conditions were analyzed with paired sample *t* tests and effect sizes were calculated as Cohen’s *d*. In addition to these main analyses, a post-hoc analysis was performed on the estimations for individual object sizes. We used a 2 × 2 × 3 ANOVA (body size x synchronicity x object size) that tested whether the perceptual effects found in the main analysis were driven by a particular object size, which we expected should not be the case. In addition, we performed a 2 × 2 ANOVA for each object size.

Although we were most interested in the perceptual change within participants as a percentage of their individual mean, we also included an analysis of the raw data (after normalizing all object size estimations to the 30 cm object) to ensure that the found effects were robust and did not depend on the transformation of the data described above. After ensuring normality with Kolmogorov–Smirnov tests we performed the same analysis as for the transformed data.

For each statement on the questionnaire, we compared the ratings from the synchronous conditions with the ratings from the asynchronous conditions. We used nonparametric Wilcoxon signed rank tests to compare conditions and we calculated the associated effect sizes as *r* for nonparametric tests.

In addition to these within-subject analyses of object size estimation and the illusion-questionnaire, we performed a between-subject correlation analysis to investigate the relation between these two measures. We hypothesized that the participants who experienced stronger invisible body illusions would have a stronger effect on object size estimation. To this end, we calculated participants’ individual illusion-score and their individual perceptual effect. We defined the illusion-score as the mean rating of illusion statements minus the mean rating of control statements, for both synchronous conditions. The perceptual effect was defined as the interaction term (in cm) of the four experimental conditions ((small-synchronous – small-asynchronous) – (large-synchronous – large-asynchronous)) divided by the average (in cm) of all four conditions. Thus, participants’ perceptual effect was relative to their individual overall mean, which improves the accuracy of between-subject analyses by eliminating the part of inter-subject variability that is independent of our experimental manipulations (see above). We used a non-parametric Spearman correlation analysis to test our hypothesis. Post-hoc, we tested which of the illusion statements contributed to the main correlation analysis by performing a Spearman correlation between each of the illusion statements and the perceptual effect. We corrected the resulting p-values with a Bonferroni correction for multiple comparisons.

## Additional Information

**How to cite this article**: van der Hoort, B. and Ehrsson, H. H. Illusions of having small or large invisible bodies influence visual perception of object size. *Sci. Rep.* **6**, 34530; doi: 10.1038/srep34530 (2016).
